# Supplementary material for: G9a/GLP inhibition during ex vivo lymphocyte expansion increases in vivo cytotoxicity of engineered T cells against hepatocellular carcinoma
Source: Nat Commun. 2023 Feb 2;14:563. doi: 10.1038/s41467-023-36160-5 (PMC9894856; doi:10.1038/s41467-023-36160-5)
Supplement: Supplementary file 1 — Supplementary Information [file 41467_2023_36160_MOESM1_ESM.pdf]

SUPPLEMENTARY FIGURES

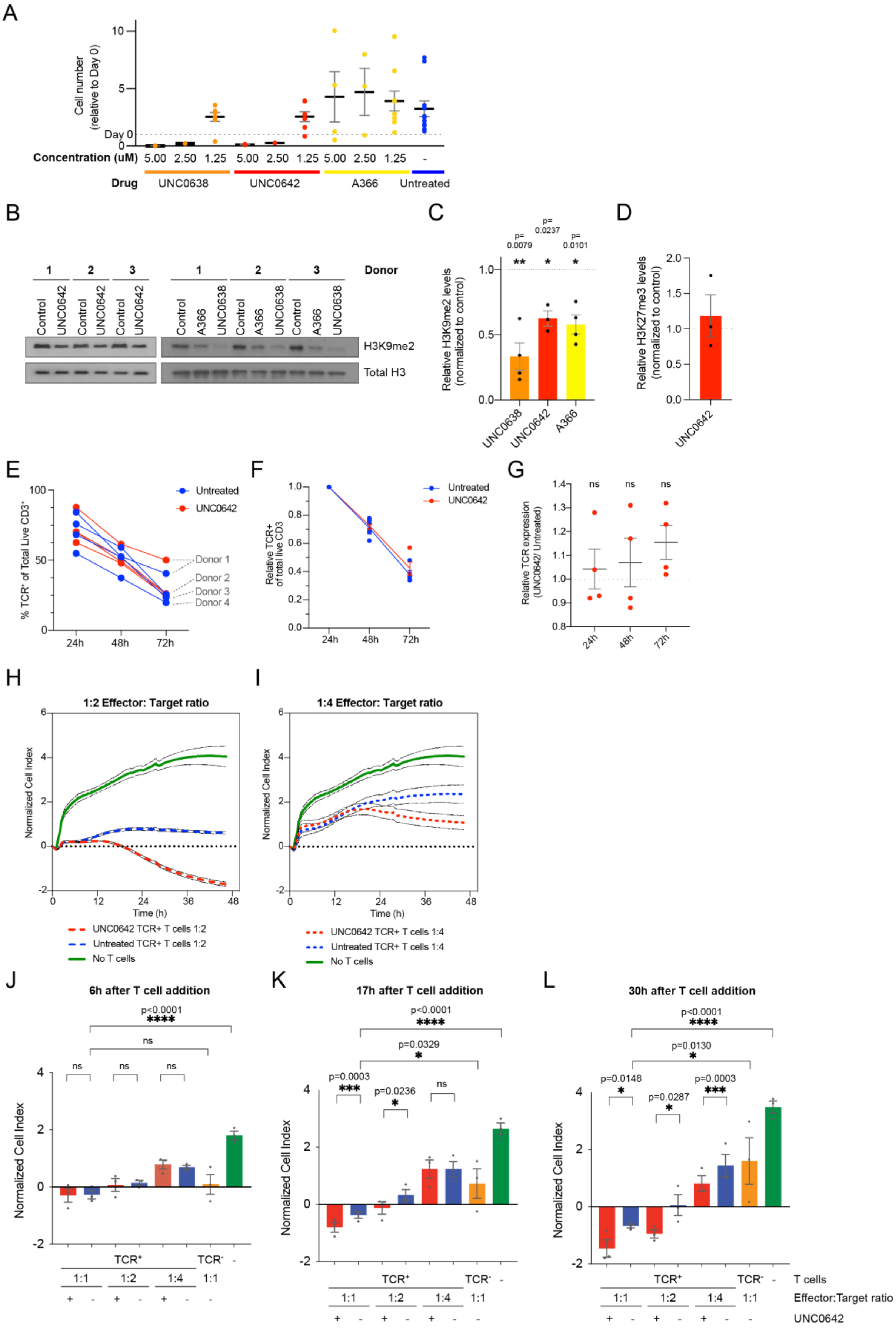
A
Cell number (relative to Day 0)
Day 0
Concentration (uM) 5.00 2.50 1.25 5.00 2.50 1.25 5.00 2.50 1.25 -
Drug UNC0638 UNC0642 A366 Untreated
B
Donor
1 2 3
Control UNC0642 Control UNC0642 Control UNC0642
Control A366 UNC0638 Control A366 UNC0638 Control A366 UNC0638
H3K9me2
Total H3
C
Relative H3K9me2 levels (normalized to control)
p= 0.0079 **
p= 0.0237 *
p= 0.0101 *
UNC0638 UNC0642 A366
D
Relative H3K27me3 levels (normalized to control)
UNC0642
E
% TCR+ of Total Live CD3+
Untreated
UNC0642
Donor 1
Donor 2
Donor 3
Donor 4
24h 48h 72h
F
Relative TCR+ of total live CD3
Untreated
UNC0642
24h 48h 72h
G
Relative TCR expression (UNC0642/ Untreated)
ns ns ns
24h 48h 72h
H
1:2 Effector: Target ratio
Normalized Cell Index
Time (h)
UNC0642 TCR+ T cells 1:2
Untreated TCR+ T cells 1:2
No T cells
I
1:4 Effector: Target ratio
Normalized Cell Index
Time (h)
UNC0642 TCR+ T cells 1:4
Untreated TCR+ T cells 1:4
No T cells
J
6h after T cell addition
p<0.0001 ****
ns
Normalized Cell Index
TCR+ TCR- -
1:1 1:2 1:4 1:1
+ - + - + - -
K
17h after T cell addition
p<0.0001 ****
p=0.0329 *
p=0.0003 ***
p=0.0236 *
ns
Normalized Cell Index
TCR+ TCR- -
1:1 1:2 1:4 1:1
+ - + - + - -
L
30h after T cell addition
p<0.0001 ****
p=0.0130 *
p=0.0148 *
p=0.0287 *
p=0.0003 ***
Normalized Cell Index
TCR+ TCR- -
1:1 1:2 1:4 1:1
+ - + - + - -
T cells
Effector:Target ratio
UNC0642

Supplementary Figure 1: G9a/GLP inhibitor effect on cell viability and H3K9me2. (A) T cell numbers after 5 days of drug treatment. Data are obtained from 4 different donors and shown as fold-change relative to cell numbers at Day 0. UNC0638 and UNC0642 exhibited cell toxicity at 2.5μM and 5.0μM. **(B)** Western blot analysis for H3K9me2 and total H3 levels for T cells from 3 different donors after UNC0642, A366 and UNC0638 treatment at 1.25μM for 5 days. **(C)** Quantification of (B). H3K9me2 levels were normalized to total H3 levels, and the data shown are normalized H3K9me2 levels in drug treated samples relative to untreated samples. Data are analysed with one-sample Wilcoxon test against a hypothetical value of 1. N = 4 biologically independent donors. **(D)** Quantification for H3K27me3 levels by Western blot for T cells from 3 different donors after UNC0642 treatment. H3K27me3 levels were normalized to total H3 levels, and the data shown are normalized H3K27me3 levels in drug treated samples relative to untreated samples. Data are analysed with one-sample Wilcoxon test against a hypothetical value of 1. N = 3 biologically independent donors. Data are shown as mean +/- SEM. **(E)** TCR expression in untreated and treated TCR^+ T cells over time. Data are shown as a percentage of CD3+ T cells. N = 4 biologically independent donors. **(F)** TCR expression in untreated and treated TCR^+ T cells from (E) normalised to 24 hours after transduction. N = 4 biologically independent donors. **(G)** TCR expression in treated TCR^+ T cells. Data shown are normalised to paired untreated samples at each time point from (F). Data are analysed with one-sample Wilcoxon test against a hypothetical value of 1. N = 4 biologically independent donors. **(H)** Example plot of normalised target cell index after addition of untreated and UNC0642-treated engineered TCR^+ T cells at 1:2 effector: target ratio and **(I)** 1:4 effector: target ratio. **(J)** Quantification of normalised target cell index at 6-hours after T cell addition, **(K)** 17-hours after T cell addition and **(L)** 30-hours after T cell addition. Data shown is for 3 different donor T cells. Data are analysed with two-way ANOVA with multiple comparison and matching within donors. All error bars represent mean and

standard error of the mean. All data are shown as mean +/- SEM. *P < 0.05, **P < 0.01, ***P < 0.001, ****P < 0.0001. Source data are provided as a Source Data file.

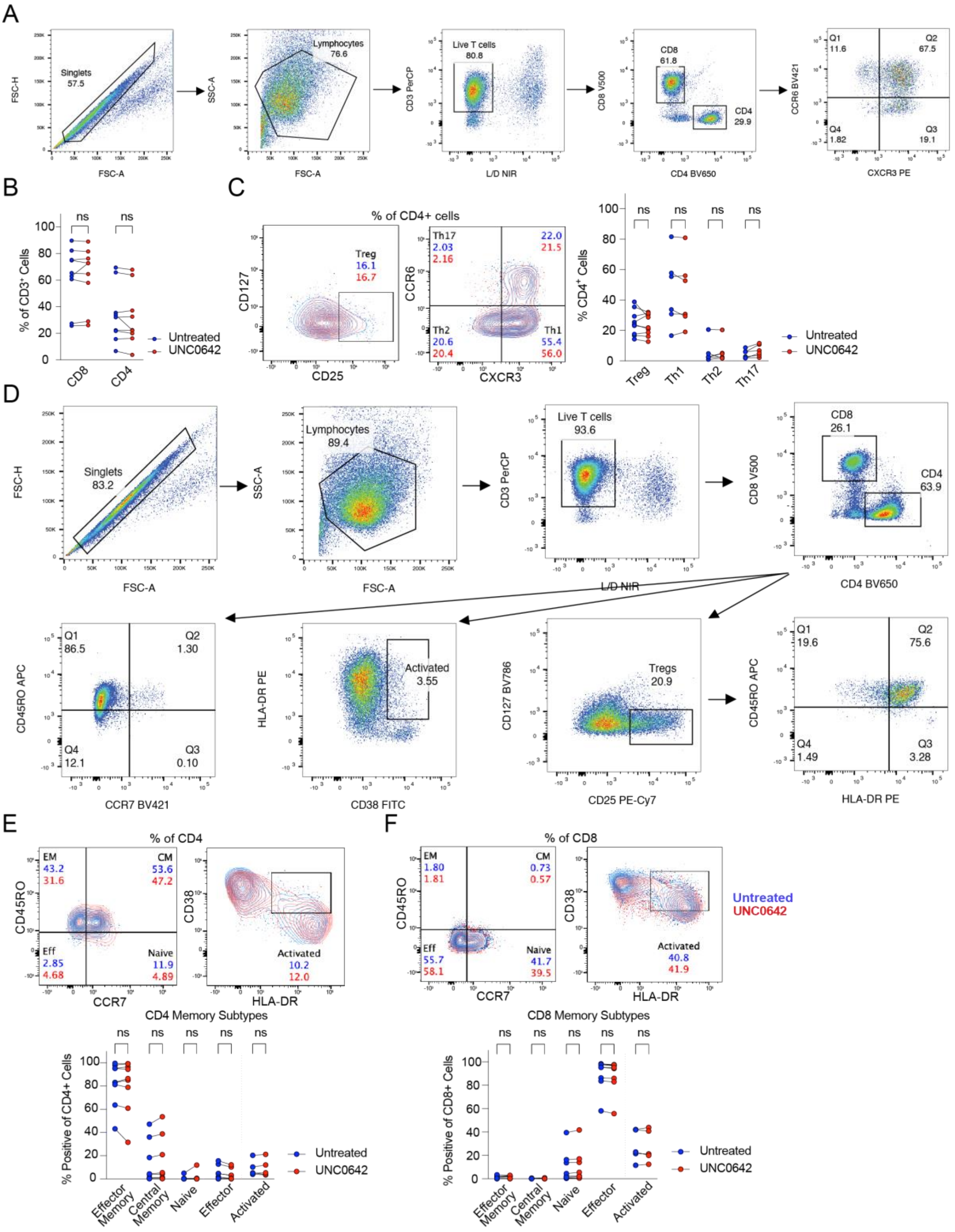

Supplementary Figure 2: Effect of UNC0642 treatment on CD4 and CD8 subtypes. (A) Flow cytometry gating strategy for CD8 and CD4 subtypes. **(B)** Percentage of CD4$^+$ and CD8$^+$ T cells with and without UNC0642 treatment. Data are shown for 9 different donors, and as a

percentage of $CD3^{+}$ T cells. **(C)** Flow cytometry characterisation of CD4+ T cell subtypes comparing untreated (blue) against UNC0642-treated T cells (red) showing Tregs and T helper cells. **(D)** Gating strategy for flow cytometry characterisation of **(E)** CD4+ T cell memory subtypes, and **(F)** CD8+ T cell memory subtypes. Data are analysed with two-way ANOVA with multiple comparison and matching within donors. N > 5 biologically independent donors, each data point represents a separate donor. All data are shown as mean +/- SEM. *P < 0.05, **P < 0.01, ***P < 0.001, ****P < 0.0001. Source data are provided as a Source Data file.

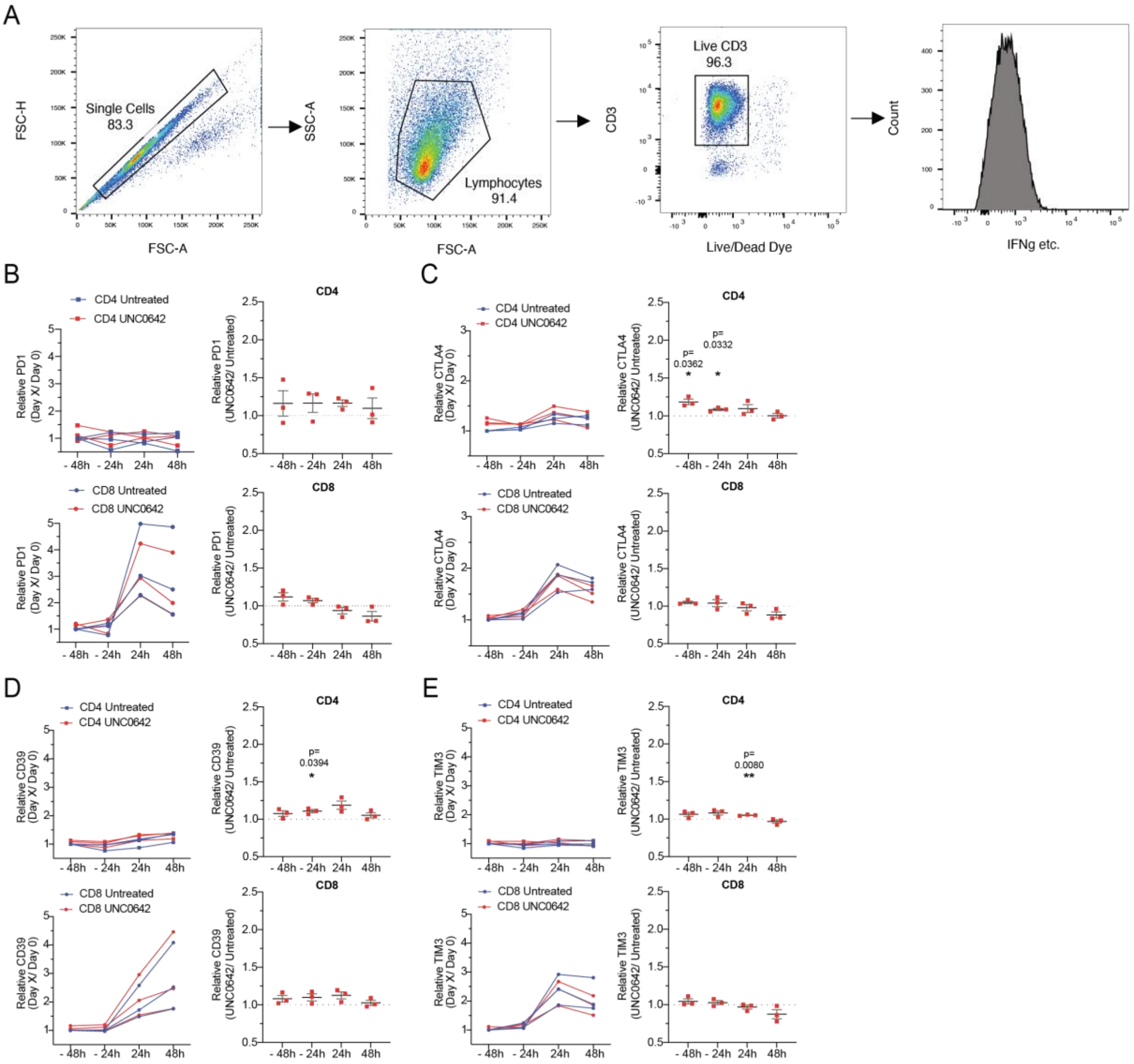

Supplementary Figure 3: Effect of UNC0642 treatment on CD4 and CD8 cell exhaustion after co-culture with target cells. (A) Flow cytometry gating strategy for exhaustion markers. **(B)** PD1 expression in untreated and UNC0642-treated TCR$^+$ CD4$^+$ (top panel) and CD8$^+$ (bottom panel) T cells over time in co-culture with HepG2-preS1. Left panel: Data are normalised to untreated sample before TCR transfection at -48 h. Right panel: Data are normalised to untreated sample at each timepoint. N = 3 biologically independent donors, each data point represents a separate donor. **(C)** CTLA4 expression in untreated and UNC0642-treated TCR$^+$ CD4$^+$ (top panel) and CD8$^+$ (bottom panel) T cells over time in co-culture with HepG2-preS1. N = 3 biologically independent donors, each data point represents a separate

donor. **(D)** CD39 expression in untreated and UNC0642-treated TCR$^+$ CD4$^+$ (top panel) and CD8$^+$ (bottom panel) T cells over time in co-culture with HepG2-preS1. N = 3 biologically independent donors, each data point represents a separate donor. **(E)** TIM3 expression in untreated and UNC0642-treated TCR$^+$ CD4$^+$ (top panel) and CD8$^+$ (bottom panel) T cells over time in co-culture with HepG2-preS1. Data are analysed with one-sample Wilcoxon ranked test against a hypothetical value of 1. N = 3 biologically independent donors, each data point represents a separate donor. All data are shown as mean +/- SEM. *P < 0.05, **P < 0.01, ***P < 0.001, ****P < 0.0001. Source data are provided as a Source Data file.

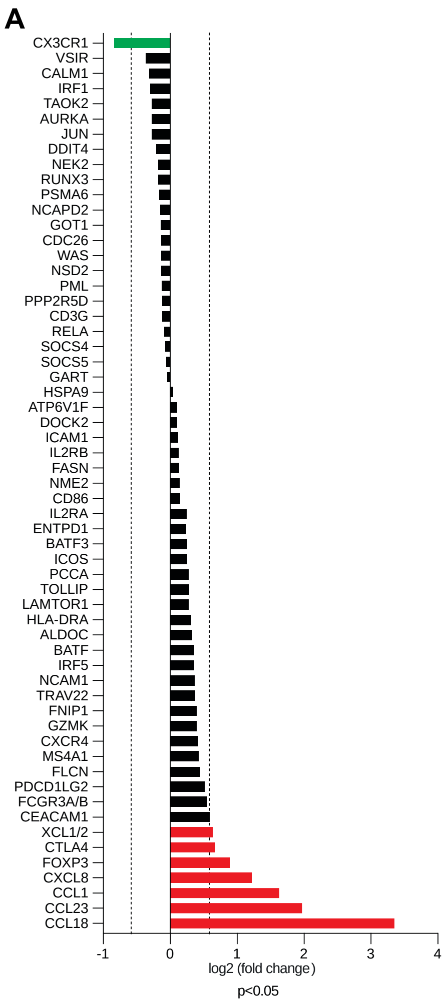

Supplementary Figure 4: Gene expression changes after UNC0642 treatment assayed using Nanostring CAR-T characterisation panel. (A) Genes with significant ($p < 0.05$) change in expression for T cells after drug treatment. Genes with a fold-change greater than 1.5 are in red, and less than 1.5 in green. Source data are provided as a Source Data file.

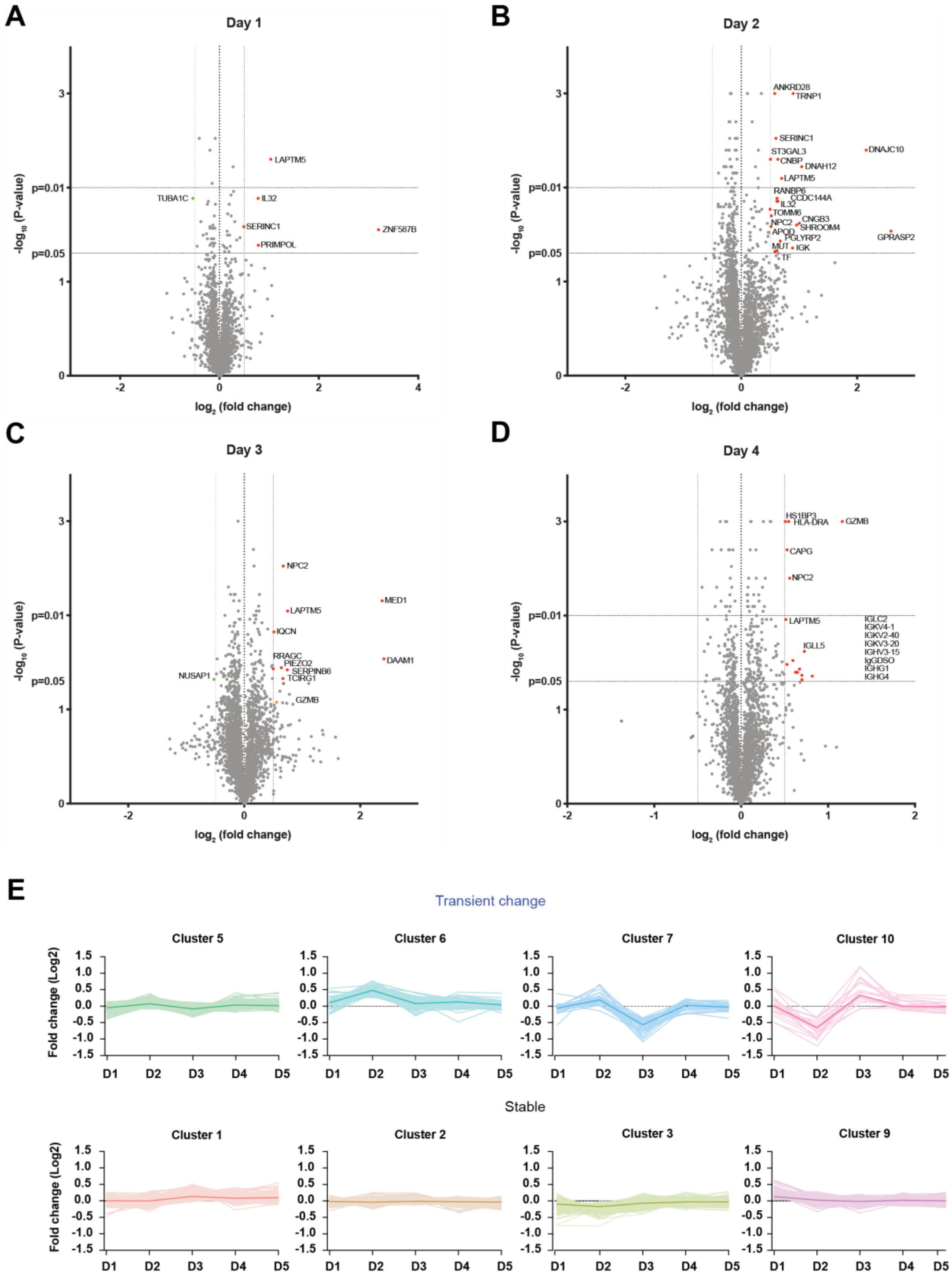

Supplementary Figure 5: Changes in protein abundance in T cells during UNC0642 treatment measured with TMT labelling. Volcano plots of changes in protein abundance of T cells after **(A)** 1 day, **(B)** 2 days, **(C)** 3 days and **(D)** 4 days of UNC0642 drug treatment (n=3

donors). **(E)** Proteins grouped by trends in changes in abundance over time by unsupervised k-means clustering. Data are represented on a plot showing fold-change over time. All data points are plotted and dark lines indicate mean for each group. Source data are provided as a Source Data file.

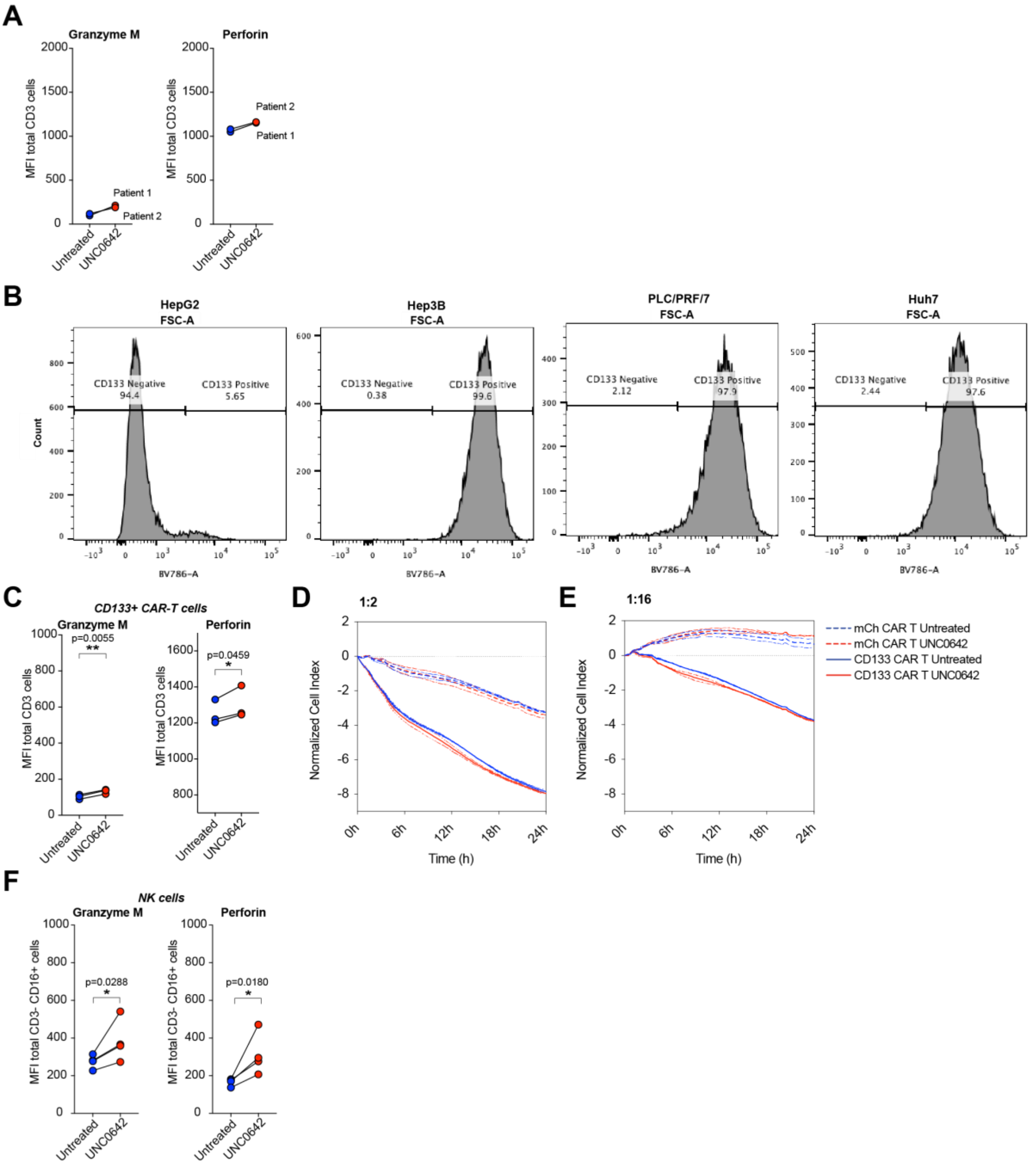

Supplementary Figure 6: CD133 expression on liver cell lines. (A) Granzyme M and perforin expression in patient-derived engineered TCR$^+$ T cells with and without UNC0642 treatment. **(B)** CD133 expression on HCC cell lines HepG2, Hep3B, PLC/PRF/7 and Huh7. **(C)** Granzyme M and perforin expression in CD133$^+$ CAR-T cells with and without UNC0642 treatment. **(D)** Example plot of normalised target cell index after addition of untreated and UNC0642-treated CD133$^+$ CAR-T cells at 1:2 effector: target ratio and **(I)** 1:16 effector: target

ratio. **(F)** Granzyme M and perforin expression in NK cells with and without UNC0642 treatment. *P < 0.05, **P < 0.01. Source data are provided as a Source Data file.

SUPPLEMENTARY TABLE

Reagent or Resource	Dilution	Source	Identifier/Catalog #
CD3 APC-Cy7	3:50	BD	557832
CD3 PerCP	3:50	BD	552851
CD4 BV650	0.625:50	BD	563875
CD8 PerCP-Cy5.5	3:50	BD	565310
CD8 V500	3:50	BD	560774
CD25 PE-Cy7	3:50	BD	557741
CD38 FITC	3:50	eBioscience	11-0389-42
CD39 APC	3:50	eBioscience	17-0399-42
CD45RO APC	3:50	BD	559865
CD56 APC	3:50	BD	555518
CD127 BV786	3:50	BD	563324
CD183 (CXCR3) PE	3:50	Miltenyi Biotec	130-120-452
CD196 (CCR6) BV421	3:50	BD	562515
CD197 (CCR7) BV421	3:50	BD	562555
CD152 (CTLA-4) PE-Cy7	3:50	eBioscience	25-1529-42
CD223 (LAG-3) PerCP	3:50	R&D Systems	FAB2319C
CD279 (PD-1) BV421	3:50	BD	562516
CD366 (TIM-3) BV711	3:50	BD	565566

Granzyme B PE	3:50	BD	561142
Granzyme M AF488	3:50	eBioscience	53-9774-41
HLA-DR PE	3:50	eBioscience	12-9956-42
IFNg PE-Cy7	3:50	BD	560741
IL-2 PerCP-Cy5.5	3:50	BD	560708
LIVE/DEAD Fixable Near-IR Dead Cell Stain Kit, for 633 or 635 nm excitation	1:1000	Invitrogen	L10119
Perforin PE	3:50	eBioscience	12-9994-42
Perforin PE-Cy7	3:50	eBioscience	25-9994-42
TCR Vβ3-FITC	5:50	Beckman Coulter	IM2372
TNF BV421	3:50	BD	562783

Supplementary Table 1: Antibodies and reagents used for flow cytometry analysis.
