# Supplementary material for: G9a/GLP inhibition during ex vivo lymphocyte expansion increases in vivo cytotoxicity of engineered T cells against hepatocellular carcinoma
Source: Nat Commun. 2023 Feb 2;14:563. doi: 10.1038/s41467-023-36160-5 (PMC9894856; doi:10.1038/s41467-023-36160-5)
Supplement: Supplementary file 3 — Reporting Summary [file 41467_2023_36160_MOESM3_ESM.pdf]

nature portfolio

Corresponding author(s): Andrea Pavesi

Last updated by author(s): Nov 14, 2022

Reporting Summary

Nature Portfolio wishes to improve the reproducibility of the work that we publish. This form provides structure for consistency and transparency in reporting. For further information on Nature Portfolio policies, see our Editorial Policies and the Editorial Policy Checklist.

Statistics

For all statistical analyses, confirm that the following items are present in the figure legend, table legend, main text, or Methods section.

n/a	Confirmed	
☐	☒	The exact sample size (*n*) for each experimental group/condition, given as a discrete number and unit of measurement
☐	☒	A statement on whether measurements were taken from distinct samples or whether the same sample was measured repeatedly
☐	☒	The statistical test(s) used AND whether they are one- or two-sided<br>*Only common tests should be described solely by name; describe more complex techniques in the Methods section.*
☒	☐	A description of all covariates tested
☐	☒	A description of any assumptions or corrections, such as tests of normality and adjustment for multiple comparisons
☐	☒	A full description of the statistical parameters including central tendency (e.g. means) or other basic estimates (e.g. regression coefficient) AND variation (e.g. standard deviation) or associated estimates of uncertainty (e.g. confidence intervals)
☐	☒	For null hypothesis testing, the test statistic (e.g. *F*, *t*, *r*) with confidence intervals, effect sizes, degrees of freedom and *P* value noted<br>*Give P values as exact values whenever suitable.*
☒	☐	For Bayesian analysis, information on the choice of priors and Markov chain Monte Carlo settings
☒	☐	For hierarchical and complex designs, identification of the appropriate level for tests and full reporting of outcomes
☒	☐	Estimates of effect sizes (e.g. Cohen's *d*, Pearson's *r*), indicating how they were calculated

Our web collection on statistics for biologists contains articles on many of the points above.

Software and code

Policy information about availability of computer code

Data collection	No software was used for data collection
Data analysis	Flojo (version 10.8). Fiji ImageJ (version 2.3). GraphPad Prism (version 9). R, K-means clustering was done using the k-means function in the stats package in R (version 4). Nanostring nCounter Advanced Analysis Software (version 2.0.134) was used for data collection of genomics.

For manuscripts utilizing custom algorithms or software that are central to the research but not yet described in published literature, software must be made available to editors and reviewers. We strongly encourage code deposition in a community repository (e.g. GitHub). See the Nature Portfolio guidelines for submitting code & software for further information.

Data

Policy information about availability of data

All manuscripts must include a data availability statement. This statement should provide the following information, where applicable:

- Accession codes, unique identifiers, or web links for publicly available datasets
- A description of any restrictions on data availability
- For clinical datasets or third party data, please ensure that the statement adheres to our policy

The data supporting the finding of this study are provided in supplement material and in the Source data file. Original image and files are also available upon reasonable request to the corresponding author.

March 2021

Human research participants

Policy information about studies involving human research participants and Sex and Gender in Research.

Reporting on sex and gender	Both patients tested were male. Gender-based analysis is not relevant for the study.
Population characteristics	Not applicable since based on compassionate grounds
Recruitment	Recruitment is based on compassionate grounds.
Ethics oversight	The treatment protocol has been approved by the SingHealth Centralised Institutional Review Board of the Singapore General Hospital where the patients were admitted and informed consent has been obtained from both patients.<br>The research was approved by the Singapore Institutional Review Board, code: NUS-IRB H17-023E<br>SINGHEALTH CENTRALISED INSTITUTIONAL REVIEW BOARD (CIRB) APPROVAL<br>CIRB Ref: 2019/2354<br>CIRB Ref: 2018/2063

Note that full information on the approval of the study protocol must also be provided in the manuscript.

Field-specific reporting

Please select the one below that is the best fit for your research. If you are not sure, read the appropriate sections before making your selection.

☒ Life sciences ☐ Behavioural & social sciences ☐ Ecological, evolutionary & environmental sciences

For a reference copy of the document with all sections, see nature.com/documents/nr-reporting-summary-flat.pdf

Life sciences study design

All studies must disclose on these points even when the disclosure is negative.

Sample size	No sample size calculation was done, as the effect of the experimental variable was unknown, however we followed the standards of the field which ensure suffcient statistics for a proper characterization of the observed phenomena. Sample sizes were typically >3 and determined by the nature of the experiment. Accordingly, only significant effect sizes in such smaller sample groups are reported in the paper.
Data exclusions	No data were excluded.
Replication	Experiments were carried out with different donors cells to account for donor-specific effects. This ensured that results were reproducible across donors. When multiple donor T cells were not feasible (i.e. for mouse experiments and patient derived T cells), reproducibility was tested by using multiple mice or repeating the experiments on different days (for patient-derived T cells). At least 3 independent replicate were performed per each experiment.
Randomization	Mouse tumours after inoculation were quntified with IVIS imaging, and equally distributed across experimental groups to ensure that each experimental group had a mix of mice with same number of "large" and "small" tumours.
Blinding	Data were not blinded, as effect of experimental variable was unknown. Where possible, analysis was automated to minimise potential bias.

Reporting for specific materials, systems and methods

We require information from authors about some types of materials, experimental systems and methods used in many studies. Here, indicate whether each material, system or method listed is relevant to your study. If you are not sure if a list item applies to your research, read the appropriate section before selecting a response.

Materials & experimental systems

n/a	Involved in the study	
☐	☒	Antibodies
☐	☒	Eukaryotic cell lines
☒	☐	Palaeontology and archaeology
☐	☒	Animals and other organisms
☒	☐	Clinical data
☒	☐	Dual use research of concern

Methods

n/a	Involved in the study	
☒	☐	ChIP-seq
☐	☒	Flow cytometry
☒	☐	MRI-based neuroimaging

Antibodies

Antibodies used	Reagent or Resource - Clone Source - Identifier/Catalog # - Dilution<br>CD3 APC-Cy7 SK7 BD 557832 3:50<br>CD3 PerCP SP34-2 BD 552851 3:50<br>CD4 BV650 SK3 BD 563875 0.625:50<br>CD8 PerCP-Cy5.5 SK1 BD 565310 3:50<br>CD8 V500 RPA-T8 BD 560774 3:50<br>CD25 PE-Cy7 M-A251 BD 557741 3:50<br>CD38 FITC HIT2 eBioscience 11-0389-42 3:50<br>CD39 APC A1 eBioscience 17-0399-42 3:50<br>CD45RO APC UCHL1 BD 559865 3:50<br>CD56 APC B159 BD 555518 3:50<br>CD127 BV786 HIL-7R-M21 BD 563324 3:50<br>CD183 (CXCR3) PE REA232 Miltenyi Biotec 130-120-452 3:50<br>CD196 (CCR6) BV421 11A9 BD 562515 3:50<br>CD197 (CCR7) BV421 150503 BD 562555 3:50<br>CD152 (CTLA-4) PE-Cy7 14D3 eBioscience 25-1529-42 3:50<br>CD223 (LAG-3) PerCP Polyclonal Goat IgG R&D Systems FAB2319C 3:50<br>CD279 (PD-1) BV421 EH12.1 BD 562516 3:50<br>CD366 (TIM-3) BV711 7D3 BD 565566 3:50<br>HBV S183 Dextramer PE HLA-A*0201 - Immudex WB3290 5:50<br>Granzyme B PE GB11 BD 561142 3:50<br>Granzyme M AF488 4B2G4 eBioscience 53-9774-41 3:50<br>HLA-DR PE LN3 eBioscience 12-9956-42 3:50<br>IFNg PE-Cy7 4S.B3 BD 560741 3:50<br>IL-2 PerCP-Cy5.5 MQ1-17H12 BD 560708 3:50<br>LIVE/DEAD™ Fixable Near-IR Dead Cell Stain Kit, for 633 or 635 nm excitation - Invitrogen L10119 1:1000<br>Perforin PE dG9 eBioscience 12-9994-42 3:50<br>Perforin PE-Cy7 dG9 eBioscience 25-9994-42 3:50<br>TCR Vβ3-FITC CH92 Beckman Coulter IM2372 5:50<br>TNFa BV421 MAb11 BD 562783 3:50<br><br>H3 Rabbit Polyclonal IgG Abcam ab1791 2ug<br>H3K9me2 mAbcam 1220 Abcam ab1220 2ug<br>H3K9me2 RM151 RevMab 31-1059-00<br>H3K27me3 Rabbit Polyclonal IgG Sigma-Aldrich 07-449
Validation	All antibodies were either validated by manufacturer or findings were validated with multiple methods as described in the material and method section

Eukaryotic cell lines

Policy information about cell lines and Sex and Gender in Research

Cell line source(s)	HEP3B cell lines were obtained by ATCC. HepG2-preS1 and HepG2-2.2.15-luc cells were shared by the lab of Antonio Bertoletti (DUKE-NUS, Singapore). PBMCs and isolated immune cells were shared by the group of Antonio Bertoletti (DUKE-NUS, Singapore) and Giulia Adriani (SigN, Singapore)
Authentication	Modified cell lines were verified with sequencing.
Mycoplasma contamination	All cell lines were tested for mycoplasma. All cell lines tested negative for mycoplasma contamination.
Commonly misidentified lines<br>(See ICLAC register)	No commonly misidentified cell lines were used in the study

Animals and other research organisms

Policy information about studies involving animals; ARRIVE guidelines recommended for reporting animal research, and Sex and Gender in Research

Laboratory animals	NOD-scid Il2rgnull (NSG) mice were purchased from The Jackson Laboratory. All mice were bred and kept under pathogen-free conditions on controlled 12-h light-dark cycle. Temperatures of ~18-23oC with 40-60% humidity were maintained throughout.
Wild animals	no wild animals were used in the study.
Reporting on sex	Since inoculation of HepG2 cells in vivo and T cell therapy were not gender dependent, there was no bias in specifically choosing a particular gender. Therefore, data shown in the manuscript were accumulated from a mixture of both female and male mice indiscriminately.
Field-collected samples	no field collected samples were used in the study

Ethics oversight

Study was approved by the Institutional Animal Care and Use Committee (IACUC) of A*STAR (Biopolis, Singapore) (IACUC No. 18139)

Note that full information on the approval of the study protocol must also be provided in the manuscript.

Flow Cytometry

Plots

Confirm that:

☒ The axis labels state the marker and fluorochrome used (e.g. CD4-FITC).

☒ The axis scales are clearly visible. Include numbers along axes only for bottom left plot of group (a 'group' is an analysis of identical markers).

☒ All plots are contour plots with outliers or pseudocolor plots.

☒ A numerical value for number of cells or percentage (with statistics) is provided.

Methodology

Sample preparation

Live and dead cells were distinguished using LIVE/DEAD™ Fixable Near-IR Dead Cell Stain Kit (Invitrogen), followed by cell surface staining, and fixation with 1% formaldehyde (MP Biomedicals). If intracellular cytokine staining was required, cells were fixed and permeabilized simultaneously with BD Cytofix/CytopermTM (BD Biosciences, USA), and intracellular cytokines were stained before fixation with 1% formaldehyde. Further details on antibodies and experiments are in supplemental methods.

Instrument

CytoFLEX (Beckman Coulter) or BD LSRII Analyzer (BD BioSciences)

Software

FlowJo

Cell population abundance

For checking HBV S183-191 TCR expression on engineered T cells, at least 1 x 105 cells were stained with viability dye, CD8, and Vβ3 antibodies.
The HBV S183-191 TCR is subsequently expressed on 20 – 80% of live CD8+ cells, depending on the donor. Experiments were conducted only if at least 50% of live cells expressed the S183-191 TCR.
For other flow cytometric analyses, at least 1 x 105 cells were stained with the various panels. Expression levels of cytokine and exhaustion markers vary depending on the donor, measured by the MFI for each marker.

Gating strategy

All flow cytometry analyses begin with gating on FSC-A/FSC-H to select for single cells, followed by FSC-A/SSC-A to exclude debris.
Dead cells were excluded with Live/dead fixable staining.
Thereafter, depending on the panel, the following gating strategies were used.

HBV S183-191 TCR Expression: CD8/Vb3 to determine percentage of CD8+Vb3+ cells.

Intracellular Cytokine Staining/Exhaustion: CD3+CD8+ cells were gated and the MFI of individual marker was recorded.

T cell memory/Thelper subtype: CD3+ cells were gated, and further split into CD4+ and CD8+ cells based on their CD4 and CD8 expression respectively.
CD45RO+CCR7- was used to identify effector memory cells.
CD45RO+CCR7+ was used to identify central memory cells.
CD45RO-CCR7+ was used to identify naïve cells.
CD45RO-CCR7- was used to identify effector cells.
CD38+HLADR+ was used to identify activated cells.
CD4+CD25+CD127- was used to identify Tregs.
CD4+CXCR3+CCR6- was used to identify Th1.
CD4+CXCR3-CCR6- was used to identify Th2.
CD4+CXCR3-CCR6+ was used to identify Th17.

☒ Tick this box to confirm that a figure exemplifying the gating strategy is provided in the Supplementary Information.
